# Management of venous thrombosis in fibular free osseomusculocutaneous flaps used for mandibular reconstruction: clinical techniques and treatment considerations

**DOI:** 10.1186/1746-160X-6-8

**Published:** 2010-06-07

**Authors:** Florian G Draenert, Martin Gosau, Bilal Al Nawas

**Affiliations:** 1 Clinic for Maxillofacial Surgery, University of Mainz, Augustusplatz 2, 55131 Mainz, Germany; 2 Clinic for Maxillofacial Surgery, University Hospital Regensburg, Franz-Josef-Strauss-Allee 11, 93053 Regensburg, Germany

## Abstract

**Background:**

Mandibular reconstruction by means of fibula transplants is the standard therapy for severe bone loss after subtotal mandibulectomy. Venous failure still represents the most common complication in free flap surgery. We present the injection of heparine into the arterial pedicle as modification of the revising both anastomoses in these cases and illustrate the application with a clinical case example.

**Methods:**

Methods consist of immediate revision surgery with clot removal, heparin perfusion by direct injection in the arterial vessel of the pedicle, subsequent high dose low-molecular weight heparin therapy, and leeches. After 6 hours postoperatively, images of early flap recovery show first sings of recovery by fading livid skin color.

**Results:**

The application of this technique in a patient with venous thrombosis resulted in the complete recovery of the flap 60 hours postoperatively. Other cases achieved similar success without additional lysis Therapy or revision of the arterial anastomosis.

**Conclusion:**

Rescue of fibular flaps is possible even in patients with massive thrombosis if surgical revision is done quickly.

## Background

Mandibular and maxillary reconstruction with fibular osseomusculocutaneous free flaps represents a common procedure that is often applied in primary and secondary reconstructions of large bony defects in these areas [1,2]. A possible complication of free flap procedures is venous failure of the anastomosis [2], which demands immediate revision surgery involving clot removal and anticoagulation therapy. We avoid the reopening of the arterial anastomosis by injecting the necessary rinsing with heparin in the arterial vessel with a small syringe.

## Methods

We apply standard anti-thrombosis prophylaxis with low molecular weight heparin, for instance, Fragmin P, but do not preoperatively use any further anti-coagulatives, such as ASS or high dose heparin. Signs of venous failure after flap surgery, which becomes visible by livid skin color, represent a peracute indication for revision surgery. Therefore, nursing staff in the intensive care unit control the flap every 2 hours within the first 72 hours after initial surgery. This procedure includes visual control of the flap color, refill control by mild compression, and palpation of the flap consistence. The revision procedure includes opening of the venous anastomosis, clot removal, and flap perfusion with 3 ml heparin solution (5000 I.E./ml). This solution is injected in the pedicle artery three times, resulting in high frequency coagulation of the punctual bleeding. In this technique, the arterial anastomosis is not opened but anticoagulation is injected in the pedicle artery. The venous vessel is re-anastomozed after several minutes of continuous blood flow from the venous pedicle vessel. Post-surgical treatment includes the use of leeches applied three times a day (four to six leeches on the skin island) until return of normal skin color.

## Case report

**Figure 1:**
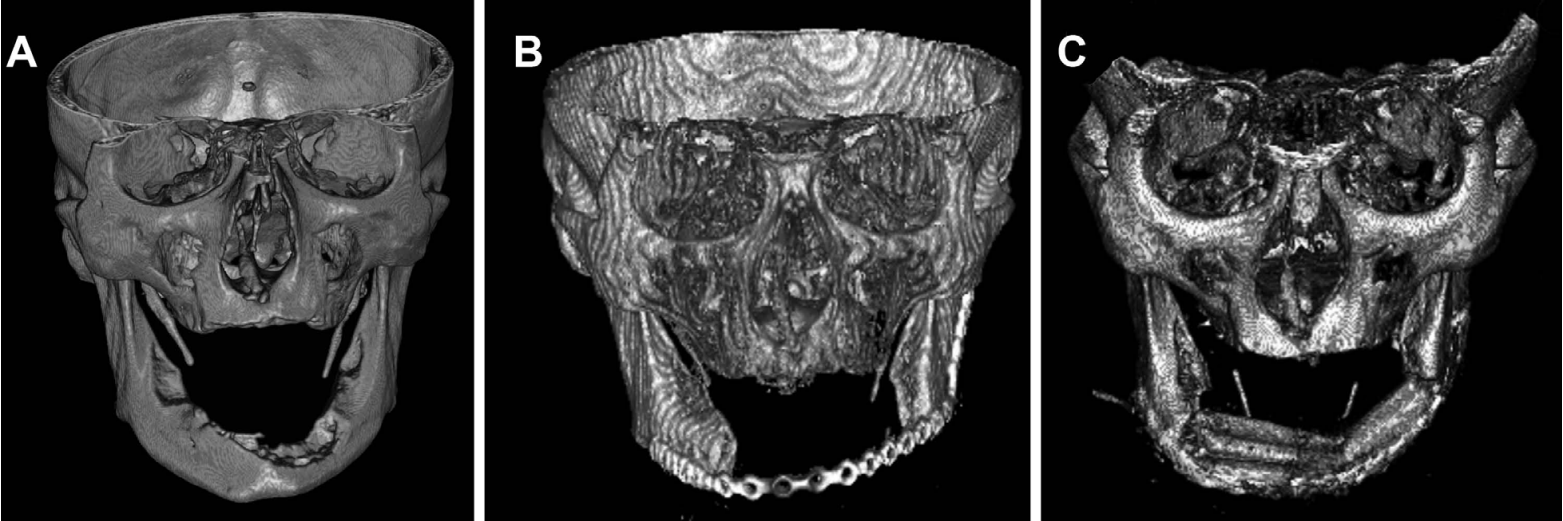
**A: 3D-CT image before tumor resection**. The infiltration of the bone is clearly visible. **B**: 3D-CT image after tumor resection with a mandibular continuity defect. **C**: 3D-CBCT after fibular osseomusculocutaneous flap reconstruction.

We report the successful clinical management of a 55-year old man with venous thrombosis of the pedicle after mandibular reconstruction by means of a osseomusculocutaneous fibular flap. Because of the diagnosis of a squamous cell carcinoma in the mandibular region in January 2007, the patient underwent hemimandibulectomy and primary soft tissue reconstruction with a radial forearm flap in combination with bilateral neck dissections (see fig. 1a and 1b). The histopathological examination showed a TNM-classification of T4a, N2c, Mx, R0, G2. After surgery, the patient underwent radiotherapy with 60 Gy, which resulted in partial necrosis of the lower lip and radiofibrosis of the surrounding soft tissue. In June 2008, the reconstruction plate perforated the epidermis and was subsequently removed.

**Figure 2:**
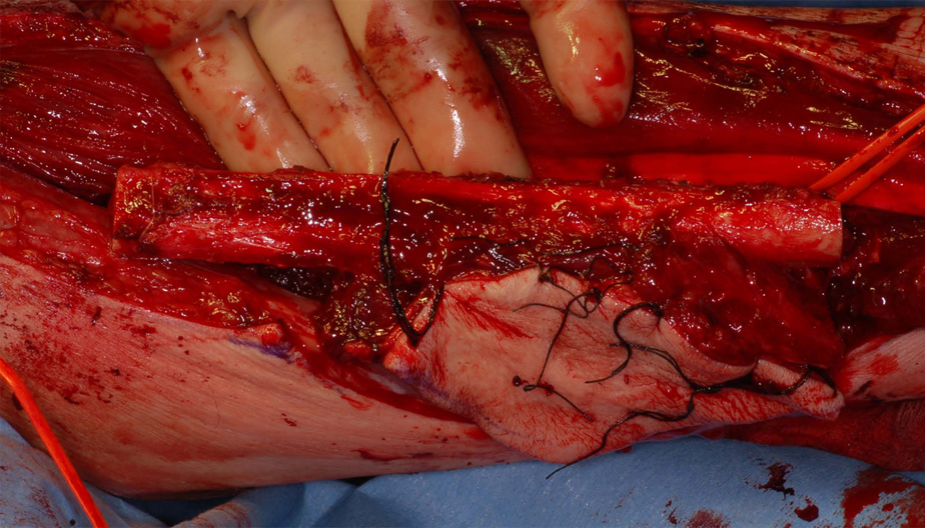
**Fibular flap harvested from the right lower limb**.

**Figure 3:**
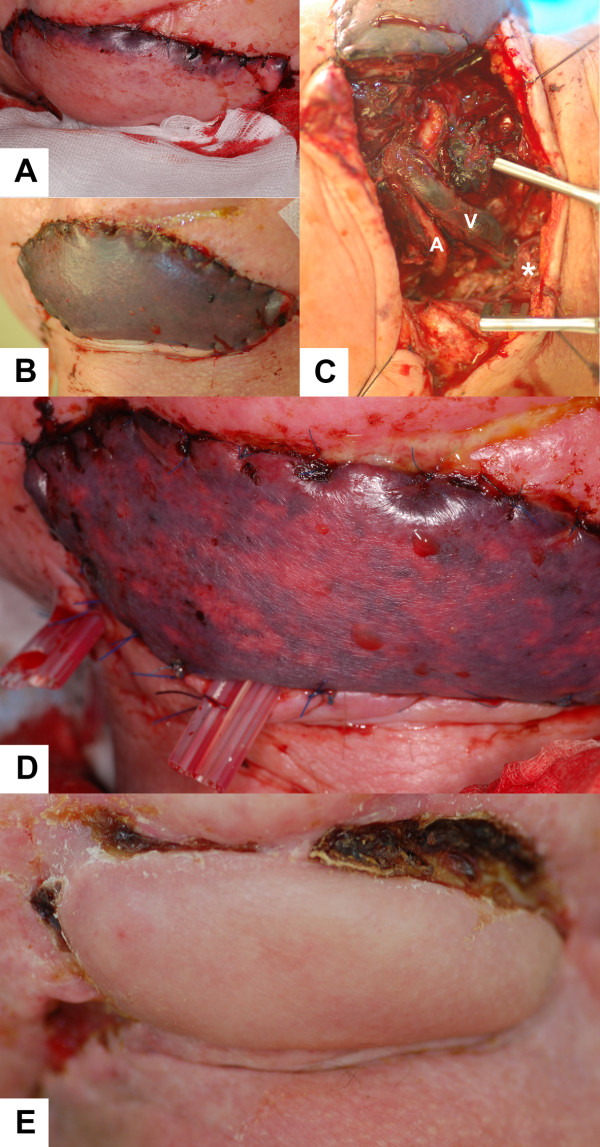
**A: 24 h after re-anastomosis**. A regular pink color of the skin island can be observed.** B**: Livid skin color after 60 hours indicates venous thrombus. **C**: The situs during revision surgery (a: artery, v: vein, star: location of venous anastomosis). **D:** Signs of venous function visible by fading livid skin color 6 hours after revision surgery. **E**: Regular wound healing and correct vessel function 5 weeks after fibular transplant surgery.

Two years after the first surgical intervention, the patient received a second mandibular reconstruction without recurrence on 4 May 2009. A fibular osseomusculocutaneous flap was harvested from the right lower limb, transplanted in the mandibular defect site, and fixed with a reconstruction plate (see fig. 1c and fig. 2). The artery was re-anastomosized to an appropriate vessel in the area of the main branch of the arteria thyroidea. Because of the lack of small vessels, venous anastomosis was done at the internal jugular vein. No complications occurred during the first 60 postoperative hours (see fig. 3a). The flap developed venous failure visible by livid skin color 60 hours after surgery (see fig. 3b). The venous part of the pedicle showed a massive thrombus at revision surgery (see fig. 3c). The clot was removed and the flap was perfused with 3 ml heparin solution (5000 I.E./ml), which was injected in the pedicle artery three times, resulting in high frequency coagulation of the punctual bleeding. The flap showed recovery of the venous function 6 hours after revision surgery detectable by the fading of the disseminated spots of livid color (see fig. 3d). The patient received Fraxiparine 0.9 mg twice per day for 2 weeks, and leeches were applied to the skin island of the flap three times per day. In the following weeks, the flap showed complete recovery with small areas of necrosis at the margins of the flap (see fig. 3e).

## Results and discussion

The described technique for treating venous thrombosis in microvascular flap surgery avoids the opening of the arterial anastomosis. This procedure has been successfully applied in several patients at the Departments for Maxillofacial Surgery of the Universities of Mainz and Regensburg as presented in this case example from Mainz (see table 1).

**Table 1:** Free fibula osseomusculocutaneous flaps with venous thrombosis treated following the described regiment.

gender	age (years)	diagnosis	reconstruction type	radiatiotherapy before flap surgery	result of revision
male	55	osteoradionecrosis	late	yes	flap survived

male	48	squamous cell carcinoma	immediate	yes	flap survived

male	46	squamous cell carcinoma	immediate	no	flap survived

male	26	Ewing sarcoma	late	no, but chemotherapy	flap lost

Late bony reconstruction after radiotherapy is still widely applied in Germany, even though early bony reconstruction has promised some advantages [3]. Patients with osteoradionecrosis and large bony defects require microvascular bony flaps, such as fibula or scapula transplants [4,5]. After radiotherapy, the number of venous vessels suitable for microsurgical re-anastomosis of the flap is often limited to jugular veins [6-8]. Compromised venous vessels in the donor region may lead to venous failure [9]. A further risk of thrombus formation is the higher prethrombotic activity in irradiated vessels [10]. Imaging techniques, such as angiography, can be applied to evaluate the vascular situation in advance [11]. We keep to the recommended practice of a minimum surveillance time of 45 min after the anastomosis of flap vessels [12].

Our described monitoring regiment includes visual control, palpation, and a manual refill test that is also described by other authors [13]. Further methods, such as a Doppler probe, are not applied [13]. Intensive care unit personnel densely control during the first 72 postoperative hours. The surgeons of our clinic additionally check the flap at least twice a day.

We immediately revise venous complications. This regiment is also described by other authors [13-16]. Local heparin injection is a well-known procedure in the management of venous thrombosis [13]. We avoid the opening of the artery by injecting high dose heparin into the pedicle artery.

Adjuvant therapy with leeches is also common practice [13,17]. Flap survival after venous thrombosis in fibula flaps is possible in most patients, but the survival rate of flaps with a bony component is lower [14]. The presented technique is one possible regiment in patients with venous thrombosis after mandibular reconstruction by means of fibular free osseomusculocutaneous flaps. We did not apply lysis therapy and never did it. However this is also a known practice with good results in several publications [18-21].

## Consent

Written informed consent was obtained from the patient for publication of this case report and accompanying images. A copy of the written consent is available for review by the Editor-in-Chief of this journal.

## Competing interests

The authors declare that they have no competing interests.

## Authors' contributions

FGD wrote the manuscript and operated the case report patient, MG documented the patients in Regensburg, BA was correcting senior author on the manuscript. All authors read and approved the final manuscript.
